# The “Prosthetic Orthodontic Approach”: An Application of the Biologically Oriented Preparation Technique Protocol

**DOI:** 10.1155/2021/5533160

**Published:** 2021-04-24

**Authors:** Luca Casula

**Affiliations:** Vita Salute University, Milan, Italy

## Abstract

In this study, three cases involving patients who required multidisciplinary treatment for the aesthetic and functional rehabilitation of the maxillary or mandibular arch are described. In particular, an indication for preprosthetic orthodontic treatment, such as orthodontic extrusion, tooth realignment, correction of malocclusion, and diastemata closure, was present in all cases. Preprosthetic orthodontic treatment to resolve these issues before the restorative procedures was proposed; however, all patients refused preprosthetic orthodontic treatment. Thus, to restore aesthetics and function, solely a feather-edge prosthetic protocol has been used. The biologically oriented preparation technique was used to prepare the teeth that were moved in the established direction by preparing the abutment more on one side than the opposite. This so called “prosthetic orthodontic approach” allowed resolving clinical issues that would typically require preprosthetic orthodontic treatment, such as complete clinical crown loss, occlusal vertical dimension loss, tooth misalignment, malocclusion, tooth agenesis, and severe multiple diastemata. The degree of reciprocal movement of the prepared teeth achievable through this approach was minor and not comparable to a traditional wide-range orthodontic movement. Besides, the technique resulted in a modification of the gingival tissues and improvement of their thickness although it is unclear what effect this technique has on the gingival biotype. None of the patients had prosthetic or periodontal complications for at least 12 months following the procedure. Gingival health was excellent, and the prosthetic procedure did not affect the pulp survival of the vital teeth. The biologically oriented preparation technique used with a prosthetic orthodontic approach can effectively manage complicated cases without the need for preprosthetic orthodontics.

## 1. Introduction

The biologically oriented preparation technique (BOPT) is a prosthetic protocol which consists of a feather-edge subgingival preparation whereby the gingival profile adapts itself to the new prosthetic coronal emergence profile [1]. This is made possible by the elimination of an emergence profile of the tooth or any preexisting finish line. A new prosthetic emergence profile is created by placing the prosthesis in such a way that leaves the gingival margin at the desired position [2, 3].

The BOPT protocol has been shown to increase gingival thickness and achieve stable and healthy soft and hard tissues in natural teeth [3, 4]. The same protocol has been described for dental implants showing good clinical outcomes [5, 6].

**Figure 1:**
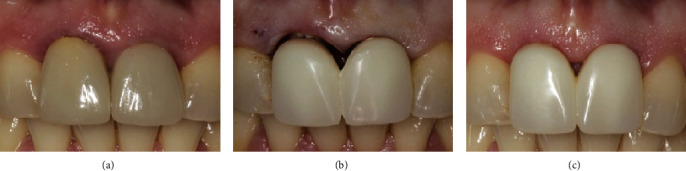
Adaptation of the soft tissue after the feather-edge preparation: (a) disparallelism of the gingival margin before BOPT; (b) right incisor shortened after BOPT; (c) coronal growth of the gingiva after one month.

**Figure 2:**
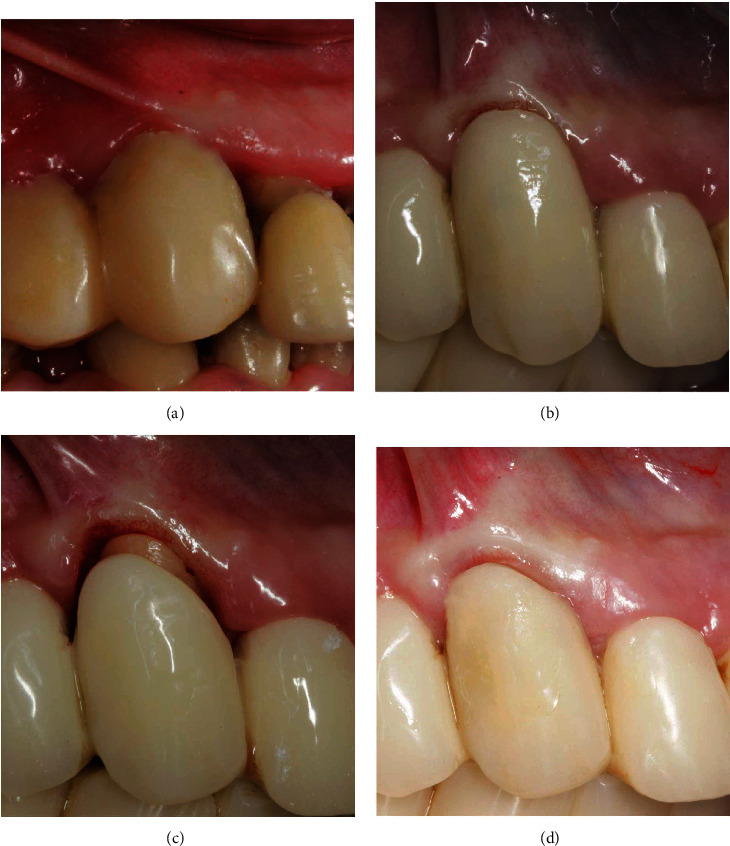
Adaptation of the soft tissue after the feather-edge preparation: (a) old complete crown restorations; (b) new interim restoration with the BOPT protocol; (c) right canine shortened after BOPT; (d) coronal growth of the gingiva after one month.

An example of soft tissue adaptation to the new prosthetic emergence profile after the BOPT protocol is shown in Figures 1 and 2. In Figure 1, the central incisor (Figure 1(a)) was prepared with a feather-edge preparation using a flame-shaped bur on the bottom of the previously probed sulcus and eliminating the preexisting finishing line. A new emergence profile was created with the interim restoration that was positioned 1.5 mm coronally to the gingival margin (Figure 1(b)). After one month of soft tissue maturation, the gingiva adapted itself to the new emergence profile (Figure 1(c)). In Figure 2, the canine (Figure 2(a)) was prepared with the same technique and the interim crown restoration was left 0.5 mm subgingivally for one month (Figure 1(b)). After this period, the interim crown restoration was shortened (Figure 1(c)), and one month later, the gingiva adapted itself to the new emergence profile (Figure 1(d)). In this series of clinical cases, the teeth were moved by means of the feather-edge preparation, avoiding a preprosthetic orthodontic treatment, and the BOPT protocol was then used to guide the soft tissue maturation to the new tooth position. This tooth movement solely by means of the dental preparation was termed the “prosthetic orthodontic approach (POA).” The approach allowed only small tooth movements and is not suitable to perform complex orthodontic movements with a large range of action. In the clinical cases described, we presented all thick gingival biotypes with the exception of the lower incisors in case number two where we also made a diagnosis of severe gingival recession. The procedural technique followed would seem to result in a thickening of the gingiva [1–6]. The phenotype after therapy appeared pink and healthy, although it is unclear what effect this technique has on the gingival biotype itself [1–6]. Further studies, both clinical and histologic, are needed to elucidate what the biological mechanisms are for gingival tissue to be modified.

## 2. Methods

The prosthetic procedure, laboratory stages, and used materials were the same for all three clinical cases described below.

**Figure 3:**
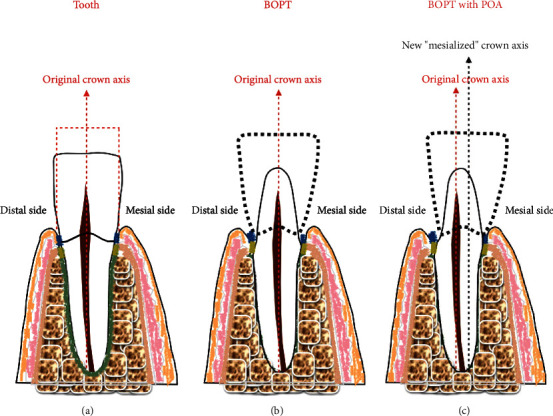
Mesialisation of the abutment tooth using the biologically oriented preparation technique (BOPT): (a) tooth; (b) the tooth is prepared without a finish line (BOPT), equally on the distal and mesial sides, such that the tooth axis remains unchanged; (c) the tooth is prepared without a finish line (BOPT), more on the distal than on the mesial side: this allows the mesialisation of the crown axis.

**Figure 4:**
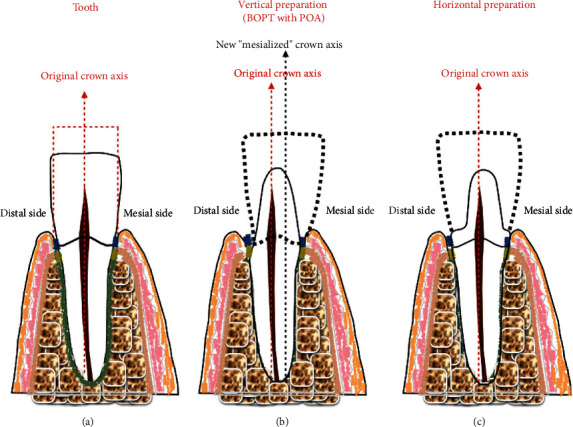
Mesialisation of the abutment tooth using the biologically oriented preparation technique (BOPT): (a) tooth; (b) the tooth is prepared without a finish line (BOPT), more on the distal than on the mesial side: this allows the mesialisation of the crown axis; (c) the tooth is prepared with a horizontal finish line, such that the crown restoration is in continuity with the external profile of the root. Therefore, the crown axis cannot be moved without altering the tooth proportions, even if the distal side is prepared more than the mesial side.

The preparation technique we used in all cases followed the BOPT protocol described by Loi in 2013. This technique involves the use of a diamond flame-shaped bur (6863D.314.016, Komet, Italy) for the abutment tooth preparation mounted in a high-speed handpiece. Before starting the dental preparation, the gingival sulcus was sounded with a calibrated probe (Hawe Click-Probe®, KerrHawe SA, Switzerland) to establish the preparation depth. The tooth was prepared until the bottom of the probed sulcus. A vertical area without a finish line was created, and the interim restoration was relined using a self-curing methacrylate resin (Sintodent, Italy). The new emergence profile was positioned in the gingival sulcus without violating the biologic width. The movement of the crown axis of the abutment teeth was achieved by means of the BOPT with the POA in all cases where tooth movement was required. The tooth movement was obtained by preparing the abutment teeth with the feather-edge preparation (BOPT) more on the one side than the opposite. For example, if mesial movement of the abutment tooth was required, the tooth was prepared more on the distal side so that the abutment tooth could be moved mesially (Figure 3). In this way, the crown could be moved in the desired direction while simultaneously maintaining the tooth proportion, simulating an orthodontic treatment. In the preparation without a finish line (vertical preparation), the crown is positioned in a vertical area of the abutment tooth so that when the vertical area is moved in a given direction, the crown can be moved together with the abutment tooth and the same tooth proportion can be maintained. Contrarily, in the horizontal finish line preparation, the crown margin is continuous with the external root profile, so that even if the distal side is prepared more than the mesial side, the crown axis cannot be moved without altering the tooth proportion (Figure 4). During the interim stage, the crown margins of the interim crown restorations were shortened if signs of inflammations (redness, bleeding) were detected, and the final impressions were taken only when the soft tissue appeared healthy and free from inflammation at every point.

### 2.1. Case 1

**Figure 5:**
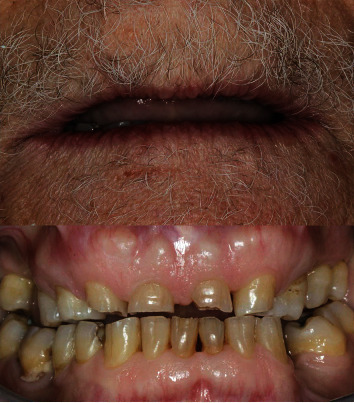
Extraoral and intraoral view of the severely worn dentition.

**Figure 6:**
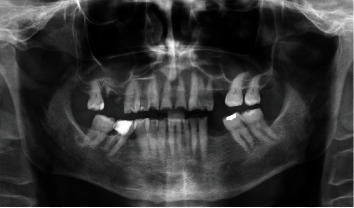
The panoramic radiograph showing the lateral incisor agenesis.

**Figure 7:**
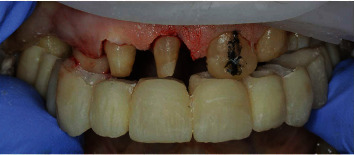
The abutment teeth were adapted to the interim restoration used as a guide for the dental preparation.

**Figure 8:**
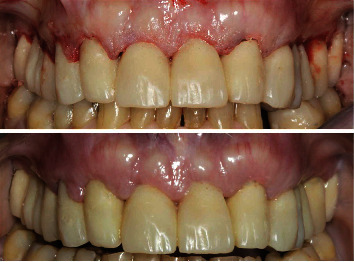
The interim restoration immediately after the prosthetic procedure and after one month of soft tissue maturation.

**Figure 9:**
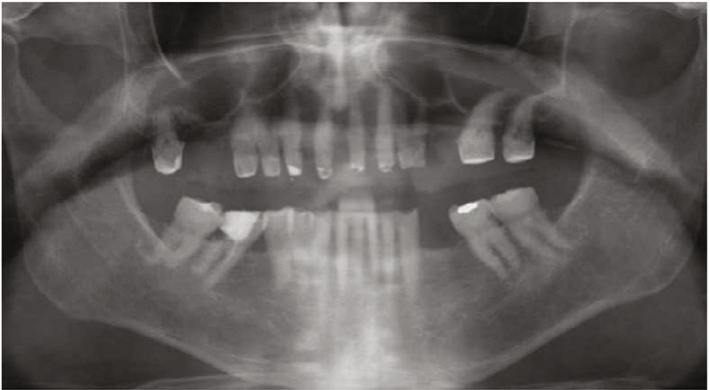
The panoramic radiograph with the interim restoration showing that the dental pulp was not involved after the prosthetic procedure.

**Figure 10:**
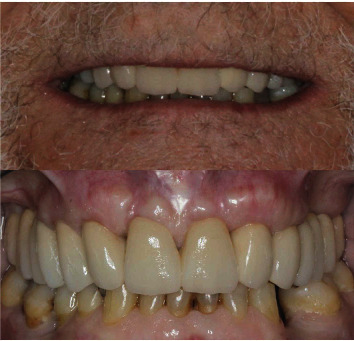
Extraoral and intraoral views of the final restoration after the 1-year follow-up.

A 71-year-old man, American Society of Anesthesiologists (ASA) type I, presented to a hospital dental department hoping to correct aesthetic and functional problems of the maxillary arch (Figure 5) where the teeth were severely worn and with the lateral incisor agenesis (Figure 6). The full-arch interim crown restoration was fabricated with the correct tooth proportion and position, in such a way that the lateral incisors replaced the misaligned canine. The correct tooth proportions were achieved by preparing the distal side of the canines more than the mesial side with the BOPT and POA, so that the abutment teeth were adapted to the interim restoration and not the opposite (Figure 7). The full-arch interim restoration was positioned in the maxillary arch with the crown margin 0.5 mm subgingivally, and after one month of soft tissue maturation, the soft tissue appeared healthy without signs of inflammation (Figure 8). A panoramic radiograph was made with the interim restoration showing that the dental pulp was not involved after the prosthetic procedure (Figure 9). The definitive impression was taken using a double retraction cord (Ultrapack™, Ultradent Products, USA) soaked in a haemostatic solution (Gengistal, Ogna, Italy) and a one-step light body and putty polysiloxane impression (Putty and Light Elite HD, Zhermack, Italy). The impression was sent to the dental technician with a request that the final restorations be shaped in a manner that allowed the emergence profile to sustain the mature soft tissues. In the dental laboratory, the final restoration was fabricated with a zirconia framework veneered with feldspathic porcelain (VITAVM 9, VITA Zahnfabrik, Germany). The final restorations were clinically checked and sent for the final glaze and at the final appointment were cemented (placed 0.5 mm into the gingival sulcus) with a self-adhesive resin cement (RelyX Unicem; 3 M ESPE, USA). At the one-year follow-up visit, no biological or technical complications were noted, and the gingiva appeared thick, pink, healthy, and completely adapted to the definitive restoration (Figure 10).

### 2.2. Case 2

**Figure 11:**
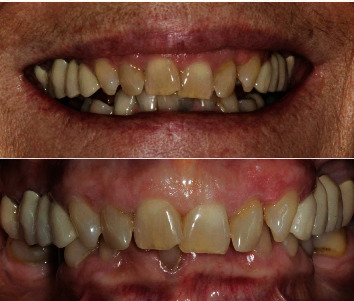
Extraoral and intraoral views showing deep bite and gummy smile.

A 70-year-old healthy woman presented at a private practice dental office, unsatisfied with her smile; this was due to excessive maxillary gingival display (a gummy smile), concomitant with short clinical crowns (Figure 11). Intraoral examination revealed partial edentulism, rotated anterior maxillary teeth with incisal erosion, and enamel wear (Figure 11). The mandibular molars were mesially inclined, resulting in the collapse of the occlusal vertical dimension (OVD) [7, 8]. The remaining mandibular teeth were severely worn. The right central incisor was rotated in the buccal position, with gingival recession and loss of gingival height (Figure 11).

During the next appointment, the anterior maxillary teeth were prepared with the BOPT, and the interim restoration was cemented. Next, two 3.8 mm diameter implants (WINSIX® BIOSAF IN Srl, Milan, Italy) were placed in the area of the mandibular first molar and left submerged during the osseointegration period. At the end of the session, two alginate impressions and a new centric relation record were obtained and mounted in an articulator to fabricate a mandibular full-arch restoration corresponding to the increased OVD.

**Figure 12:**
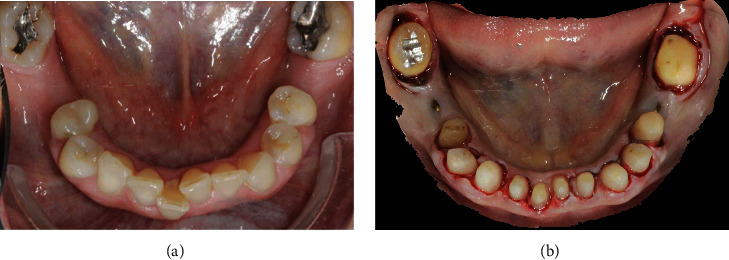
The mandibular arch showing the right central incisor and the second molars before the prosthetic preparation (a) and realignment after the POA (b).

**Figure 13:**
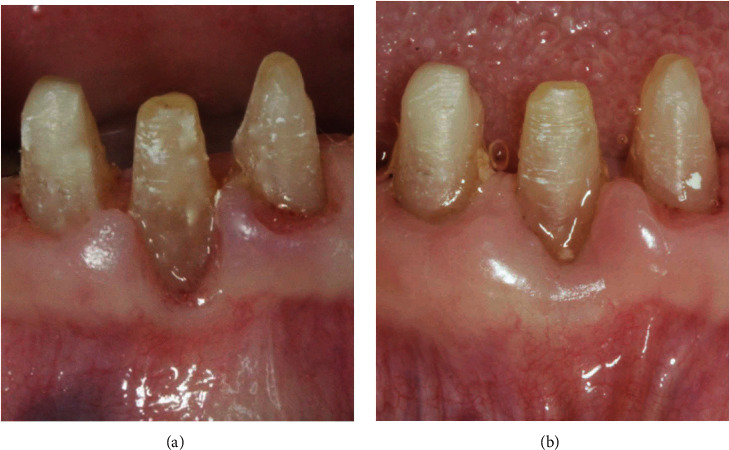
The mandibular right central incisor without the interim restoration: (a) gingival recession; (b) healing of the gingival recession and thickening of the gingiva after the BOPT protocol with POA.

**Figure 14:**
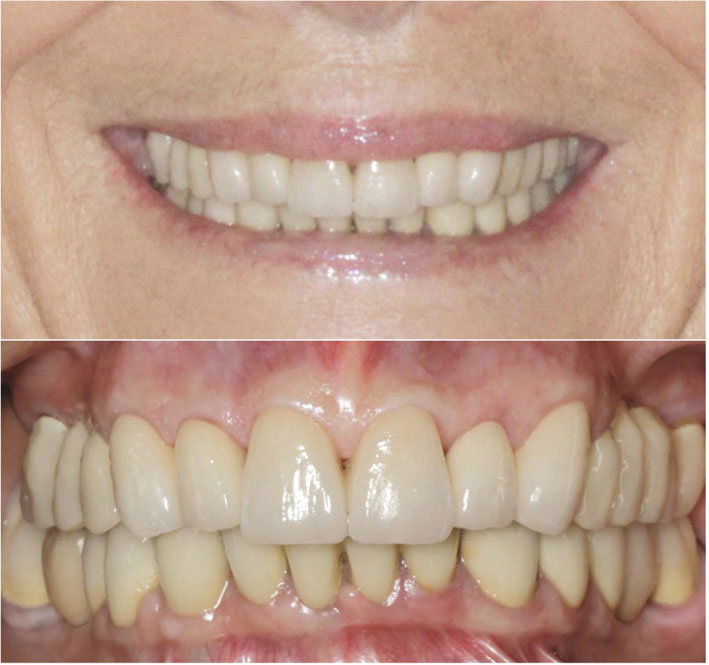
Final rehabilitation at the two-year follow-up with the new smile after the correction of the deep bite and gummy smile.

**Figure 15:**
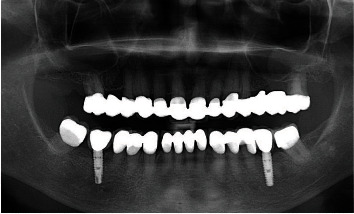
Panoramic radiograph taken two years after the procedure showing that the dental pulp was not involved after the prosthetic procedure.

The mandibular full-arch restoration was fabricated using a 3 mm OVD increment, and the curve of Spee was harmonized with the opposing arch. The mandibular teeth were prepared with the BOPT, and the complete restoration was placed in the mandibular arch from the right to the left second molar. The mandibular left second molar was mesially inclined, so it was prepared more on the mesial side than on the distal one using the BOPT with the POA to move the dental axis distally, thereby creating an adequate space for the first molar parallel to the path of prosthesis insertion (Figure 12). The POA was also used for the mandibular right central incisor by preparing it more on the vestibular side to realign it with the adjacent teeth (Figure 13). The interim mandibular full-arch restoration was cemented and corresponded to the new OVD. One month after soft tissue maturation, the definitive impression of the maxillary abutment teeth was taken, and zirconia ceramic full-crown restorations were fabricated in the dental laboratory. The gingival sulcus was probed again and found to be 2 mm on the vestibular side. The dental technician was instructed to position the crown margin 1.5 mm subgingivally only on the vestibular side, using all of the available gingival sulcus, thus improving the gummy smile (Figure 14). In the interdental spaces and on the palatal side, the crown margin was placed 0.5 mm into the gingival sulcus. The patient expressed satisfaction with the results. Healthy gingiva, with an ideal scalloped architecture, was observed at the 2-year follow-up. No gingival recession or inflammation was noted. The restoration accomplished three goals: (1) correction of the deep bite, (2) realignment of the teeth, and (3) elimination of the gummy smile. The procedure did not affect the pulp vitality, and no endodontic complications were observed at the 2-year follow-up (Figure 15).

### 2.3. Case 3

**Figure 16:**
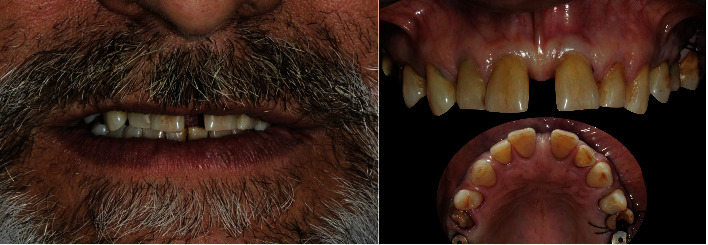
Preoperative extraoral and intraoral views showing 6 mm midline diastema.

A 63-year-old man, ASA type I, presented at a hospital dental department. The subject wanted to improve their dental aesthetics and function. Intraoral examination revealed a 6 mm maxillary midline diastema and multiple diastemata, rampant caries, radicular fractures, and periodontal involvement of the first molars, which were immediately extracted and replaced with 3.8 diameter implants (WINSIX® BIOSAF IN Srl, Milan, Italy) that were loaded 3 months after placement (Figure 16).

**Figure 17:**
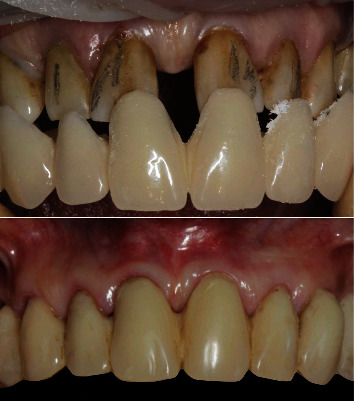
The full-arch interim crown restoration used as a guide for the dental preparation and the correct tooth proportion achieved after BOPT with POA.

The correct tooth proportions were achieved by preparing the distal side more than the mesial side with the BOPT and POA. Thus, during abutment preparation, the vertical area was moved toward the centre of the tooth, and the mesialisation of the teeth concurrently facilitated diastema closure and maintained the correct tooth proportions (Figure 17). As previously described, the interim restoration was used as a guide during tooth preparation, and the distal side of the teeth was prepared until a point previously marked with a pencil to put the interim crowns on the abutment teeth (Figure 17). The BOPT was completed, and the crown margins were positioned 0.5 mm within the gingival sulcus (Figure 17).

**Figure 18:**
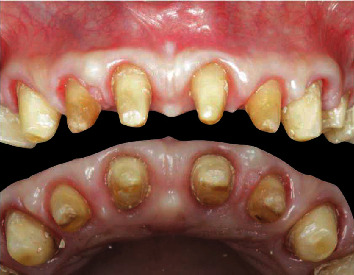
Abutment teeth 8 weeks after soft tissue maturation.

**Figure 19:**
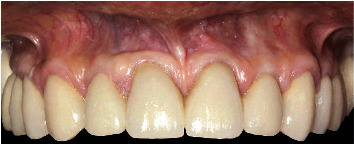
Definitive restoration 2 years after the procedure.

**Figure 20:**
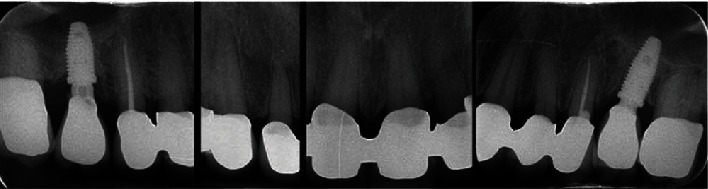
Periapical radiograph taken 24 months after the procedure.

Eight weeks after soft tissue maturation, the gingival tissues appeared healthy (Figure 18), and the definitive restorations were completed. The patient showed healthy gingiva, no probing depths more than 3 mm, no bleeding upon probing, and no signs of inflammation at the 2-year follow-up. Further, the prosthetic restorations were functionally integrated with the soft tissues, and the gingiva and interdental papillae were pink and stippled (Figure 19). Diastemata closure was achieved, and the prosthetic crowns were well proportioned. The procedure did not affect the pulp vitality, and no endodontic complications were observed at the 2-year follow-up (Figure 20).

## 3. Discussion

The BOPT provides a feather-edge subgingival preparation on natural teeth, for which good clinical periodontal outcomes and thickening of keratinized tissues are reported [1–6]. Feather-edge preparation offers a good marginal fit with similar outcomes to other designs, and it was recently reported that restoration margins located within the gingival sulcus do not cause gingivitis if the patient practices appropriate oral hygiene [9–11].

This abutment tooth preparation consists of a vertical area in which the crown margin is positioned. This vertical area can be moved in a given direction by preparing the abutment tooth more on the one side than the other, and the crown restoration is moved together, allowing the clinician to move the tooth axis in a mesio-distal or buccal-lingual direction (Figure 3). These concepts were applied in the present clinical cases, in which orthodontics would have been a primary treatment option [12–15]. However, orthodontic treatment was refused by the patients; thus, an alternative treatment plan was proposed, which comprised a prosthetic procedure using the BOPT with a POA.

This prosthetic procedure allowed for the transformation of the tooth shape and position, as is presented in the first case report in which the maxillary canines were transformed into the missing lateral incisors and the first maxillary premolars were moved mesially and transformed into canines. In the other cases, the BOPT with a POA was able to facilitate significant improvements in function and aesthetics in patients with a collapsed OVD with a limited prosthetic space, tooth misalignment, gingival recession, a gummy smile, and multiple and severe diastemata. This procedure was adopted in these cases because tooth wear and aesthetic problems were also present. The vertical area without a finish line, in contrast to the horizontal finish line, allowed clinicians to move the interim restoration in an apical or coronal direction, which greatly facilitates tissue adaptation to the emergence profile of the crown restoration [1–6]. In the second case, movement of the interim restoration allowed the gingival recession on the mandibular right central incisor to be closed (coronal movement) and corrected the gummy smile in the anterior maxillary sector (apical movement) [16]. Nevertheless, this approach is limited to cases where rehabilitation with complete clinical crown restorations is required for teeth with unsuitable clinical crown restoration or in severely worn teeth or those with aesthetic problems. A drawback to this technique is that it involves the use of crown margin restoration placed in a subgingival position that has serious implications for the health of the supporting periodontal tissues [17–19]. Inflammation of the gingival tissues was avoided by placing the crown margin only in the gingival sulcus and not in the junctional epithelium or supracrestal connective tissue [11]. This was achieved before tooth preparation by probing the gingival sulcus with the calibrated dental probe and thus deciding the preparation depth. During the dental preparation, the depth was observed by preparing the abutment tooth with a calibrated bur (graduated into millimetres). During the interim stage, tissue inflammation was avoided by shortening the crown margins of the interim crown restorations if signs of inflammation (redness, bleeding) were detected, and the final impressions were taken only when the soft tissue appeared healthy and free from inflammation. In the vertical preparation, there is no dental finish line to refer to, and it is difficult for the dental technician to place the crown margin adequately. To avoid problems related to this, the crown margin of the definitive restoration was controlled during the clinical framework examination, and if necessary, was shortened. After the framework-soft tissue relationship was captured with an impression, the dental technician who was aware of the depth rechecked all of the crown margins before veneering.

Orthodontic treatment with a multidisciplinary approach would have been recommended as the primary treatment option for all of the clinical situations described in this study; however, in cases in which a patient is unable or unwilling to undergo preprosthetic orthodontic treatment, it is possible to instead use the BOPT with a POA to simulate multidisciplinary treatment [20–24]. This approach allowed only small reciprocal tooth movements and not really extensive range orthodontic outcomes. Previous studies describe how this technique is beneficial to obtain gingival modifications especially in the presence of gingival recessions [3, 17]. Indeed, the technique is prone to affect both the gingival thickness and the height of the keratinized gingiva [1–6, 17]. Our clinical outcomes resulted in gingival thickening with acquisition of an excellent pink and healthy gingiva, and the same protocol showed good clinical outcomes for dental implants [5, 6, 25]. Further studies, both clinical and histological, are needed to clarify what mechanisms underlie the biological conditioning of gingival tissues and their biotype.

## 4. Conclusion

In the present case report, the BOPT used with a POA effectively managed complicated cases without the need for preprosthetic orthodontic treatment. The absence of a finish line in natural abutment teeth offers many possibilities to clinicians for solving situations in which a multidisciplinary approach is usually required. After at least one year, none of the patients experienced prosthetic or periodontal complications; the gingival tissues were healthy, and the odontotomies did not affect the pulp survival of the vital teeth. Nevertheless, long-term clinical studies are needed to validate the results of these cases.
